# The iSBTc/SITC primer on tumor immunology and biological therapy of cancer: a summary of the 2010 program

**DOI:** 10.1186/1479-5876-9-18

**Published:** 2011-01-31

**Authors:** James M Balwit, Patrick Hwu, Walter J Urba, Francesco M Marincola

**Affiliations:** 1 Society for Immunotherapy of Cancer, Milwaukee, WI, USA; 2 Department of Melanoma Medical Oncology, the University of Texas, MD Anderson Cancer Center, Houston, TX, USA; 3 Earle A. Chiles Research Institute, Providence Portland Medical Center, Portland, OR, USA; 4 Infectious Disease and Immunogenetics Section (IDIS), Dept. of Translation Medicine, Clinical Center, and Center for Human Immunology (CHI), National Institutes of Health, Bethesda, MD, USA

## Abstract

The Society for Immunotherapy of Cancer, SITC (formerly the International Society for Biological Therapy of Cancer, iSBTc), aims to improve cancer patient outcomes by advancing the science, development and application of biological therapy and immunotherapy. The society and its educational programs have become premier destinations for interaction and innovation in the cancer biologics community. For over a decade, the society has offered the Primer on Tumor Immunology and Biological Therapy of Cancer™ in conjunction with its Annual Scientific Meeting. This report summarizes the 2010 Primer that took place October 1, 2010 in Washington, D.C. as part of the educational offerings associated with the society's 25th anniversary. The target audience was basic and clinical investigators from academia, industry and regulatory agencies, and included clinicians, post-doctoral fellows, students, and allied health professionals. Attendees were provided a review of basic immunology and educated on the current status and most recent advances in tumor immunology and clinical/translational caner immunology. Ten prominent investigators presented on the following topics: innate immunity and inflammation; an overview of adaptive immunity; dendritic cells; tumor microenvironment; regulatory immune cells; immune monitoring; cytokines in cancer immunotherapy; immune modulating antibodies; cancer vaccines; and adoptive T cell therapy. Presentation slides, a Primer webinar and additional program information are available online on the society's website.

## Innate Immunity and Inflammation

Innate immunity and inflammation play important roles in the development and response to cancer. Willem W. Overwijk, PhD (MD Anderson Cancer Center) provided an overview of the cells and molecules involved in innate immunity, highlighting the role of inflammation in cancer. While inflammation is a classic hallmark of cancer, the outcomes following activation of innate immunity and inflammation in cancer can vary. In some cases inflammation can promote cancer; in other cases, suppress it.

Examples were reviewed whereby inflammation has been shown to promote cancer via collaboration with K-ras mutations and with HPV E6/E7 oncogenes. Moreover, reactive oxygen and nitrogen intermediates (ROI and RNI) generated during inflammation may promote mutations, which in turn can promote tumor initiation. Adding to this vicious cycle, the tumor microenvironment and mutations associated with tumors (e.g., BRAF mutations) can drive the innate response toward cancer-promoting inflammation. The following generalizations further illustrate this circular nature of the relationship between inflammation and cancer: inflammation can cause cancer; inflammation can cause mutation; mutation can cause inflammation; mutation can cause cancer; and cancer can cause inflammation.

Inflammation may also suppress cancer, as exemplified by the capacity of type I interferons (IFNs) to suppress the development of carcinogen-induced tumors, and by the tumor inflammation and intratumoral accumulation of T cells observed in response to CpG.

A number of therapies exist that are designed to block inflammatory processes that promote cancer as well as therapies that induce inflammatory processes shown to suppress cancer. Our understanding of inflammatory cells and molecules in cancer is currently limited. As we increase our understanding of the relationship between inflammation and cancer, we will be able to refine therapeutic interventions to improve cancer outcomes.

## Overview of Adaptive Immunity

Emmanuel T. Akporiaye, PhD (Robert W. Franz Cancer Research Center, Earle A. Chiles Research Institute, Providence Cancer Center) provided an overview of adaptive immunity with a focus on the T cell response. He illustrated the key characteristics that distinguish adaptive and innate immunity and summarized the mechanisms of T and B cell activation.

Dr. Akporiaye demonstrated how class I and class II MHC molecules on antigen presenting cells (APCs) differ in molecular structure and how this dictates peptide loading and interaction with CD4 and CD8 molecules on T cell subsets (i.e., CD8 interacts with MHC class I molecules; CD4 with class II molecules). He summarized the model in which the fate of T lymphocytes is directed by the conditions of engagement of the T cell receptor (TCR). In the "standard model," two signals are required to drive T cell activation, proliferation and differentiation to effector T cells. The first signal is the engagement of the TCR by the appropriate peptide-loaded MHC molecule. The second (co-stimulatory) signal is mediated by interaction between CD28 on the T cell and CD80/86 (B7) on the APC. Engagement of the TCR in the absence of this co-stimulatory signal drives the T cells to anergy and apoptosis. When CD80/86 binds the T cell molecule CTLA-4 during engagement of the TCR, an inhibitory signal is delivered to the activated T cell, arresting the cell cycle, serving to regulate the proliferative response of antigen-specific T cells. The binding of these molecules occurs in the immunological synapse between the T cell and APC, where clustering of molecules essential to T cell activation has been observed. This creates a narrow space for efficient delivery of effector molecules, reorients the secretory apparatus, and helps focus the T cell on its antigen-specific target.

Dr. Akporiaye presented examples of antigen peptide processing, loading and presentation within class I and II MHC molecules. Differences in the pathways were noted. In the MHC class I presentation pathway, endogenous protein antigens are degraded in the cytosol by the proteasome; the resulting peptides are transported back into the endoplasmic reticulum via the transporter associated with antigen presentation (TAP) complex. The peptides are then loaded onto newly synthesized MHC class I molecules and transported through the Golgi to the cell surface (direct presentation). Class I MHC molecules may also be loaded with peptides of exogenous origin by cross-presentation. In this case phagocytosed proteins are retrotransported out of the phagosome to the cytosol where they are degraded. The exogenous peptides are redelivered to the phagosome by the TAP complex and loaded on MHC class I molecules for transport and expression on the cell surface.

By contrast, processing of exogenous protein antigens in the MHC class II pathway occurs within acidified endosomes. The resulting exogenous peptides compete for the binding cleft with a peptide fragment (CLIP) from an endogenous molecule (invariant chain) that targets the class II molecule to the acidified vesicle. This competitive binding helps ensure loading of high avidity antigen peptides.

Dr. Akporiaye reviewed mechanisms of killing by activated cytotoxic T lymphocytes (CTLs), including the roles of the pore-forming protein perforin, which aids delivery of toxic granules to the cytoplasm of the target cells, granzymes, serine proteases that activate apoptosis, granulysin, tumor necrosis factor alpha (TNFα) and Fas-Fas ligand interactions.

In a brief overview of the B cell response, Dr. Akporiaye noted differences in how B cells and T cells recognize antigens. In contrast to T cells, B cells recognize antigen via surface immunoglobulin (Ig) and this binding is independent of MHC. Thus B cells can recognize soluble, unprocessed antigens. Whereas the epitopes recognized by T cells are sequential and linear due to the physical requirements of the MHC molecules' binding cleft, the epitope recognized by B cells may be non-sequential (or sequential). While epitopes for T cells are usually internal, B cells can recognize accessible (external) hydrophilic epitopes.

The primary antibody response to an antigenic challenge is characterized by a short lag in production of specific antibodies, followed by an extended plateau in specific IgM production and then a slow decline in titer. After a subsequent challenge, a secondary response is characterized by a rapid 10- to 1,000-fold increase in specific antibodies of the IgG class with greater affinity for the antigen than those antibodies generated in the primary response. Dr. Akporiaye provided a brief overview of the structural features of Igs and their effector mechanisms, including neutralization of pathogens and toxins, opsonization to promote phagocytosis, and complement activation.

## Dendritic Cells

Karolina Palucka, MD, PhD (Baylor Institute for Immunology Research) reviewed the biology and clinical application of dendritic cells (DCs), noting that the next generation cancer vaccines will be based on DCs reprogramming the immune system. DCs are essential for capturing, processing and presenting antigens and play a central role in attracting T cells via chemokines and regulating their differentiation. She indicated that a DC vaccine should: 1) induce high avidity CTLs; 2) induce long-term memory CD4^+ ^and CD8^+ ^T cells; 3) not induce regulatory T cells; and 4) induce CD4^+ ^T cells that help CD8^+ ^T cells. Therapeutic DC vaccine strategies have included both *ex vivo *strategies, in which immature DCs are removed, loaded with the antigen, activated and infused back to the patient, as well as strategies in which the DCs are targeted *in vivo*. First generation DC vaccines have helped to define important parameters surrounding antigen loading, which cytokines to use, how to deliver the vaccine, and how to assess the immune response following DC vaccination.

Dr. Palucka demonstrated that both short (9-10 amino acid) peptides and killed allogeneic tumor cells can induce a response. Moreover, DC vaccines can expand long-lived, polyfunctional, antigen-specific CD8^+ ^and CD4^+ ^T cells. Since patients with metastatic melanoma display tumor antigen-specific, IL-10-producing T regulatory cells (Tregs), Dr. Palucka queried whether IL-10- producing Tregs could be reprogrammed to become effector cells by DC vaccines.

Subsets of DCs demonstrate functional differences. For example, interstitial (dermal) DCs secrete IL-10 and enhance B cell differentiation, while Langerhans cells do not. Further, Langerhans cells are more efficient than interstitial DCs at priming CD8^+ ^T cells. Moreover, priming of CD8^+ ^T cells by Langerhans cells is associated with enhanced expression of the effector molecules granzyme A, granzyme B and perforin, whereas priming with interstitial DCs is only associated with granzyme B expression. The differences in the priming efficiency between these two DC subsets may be due to differences in IL-15 expression. While IL-15 is normally surface expressed, addition of free IL-15 to interstitial DCs improves their priming efficiency. These two functionally different DC subsets mediate distinct immune responses: Langerhans cells promote cellular immunity through priming of CD8^+ ^T cells mediated by IL-15, while interstitial DCs promote humoral immunity through direct priming of B cells (and indirectly by priming CD4^+ ^follicular helper T cells) mediated by IL-12. These functional differences represent potential variables to manipulate in DC vaccine strategies to promote the desired immune response.

Since different DC subsets generate distinct immune responses, the various surface molecules on DCs may represent targets with potentially distinct cellular and immune effects. Using fusion proteins composed of an antigen linked to an antibody that is directed at a particular DC surface receptor, it has been shown that targeting DCs via distinct lectins promotes distinct effects on T cell proliferation, suppression (via IL-10) and antigen-specific secretion of cytokines from T cells primed by these DCs. Thus, in addition to different DC subsets that elicit distinct immune responses, the particular surface molecule that is targeted on a given DC likewise elicits a distinct response, demonstrating functional plasticity of DCs.

Evaluation of primary breast cancer tumors reveals many CD4^+ ^T cells in close proximity to mature DC. The tumor-infiltrating T cells produce high levels of type 2 cytokines, in particular IL-13 (but not IL-10). Moreover, tissue staining demonstrates that much of the IL-13 is localized on the surface of the cancer cells. Furthermore, STAT6--a signal transducer downstream in the IL-13 receptor signaling pathway--is phosphorylated, suggesting the IL-13 from infiltrating CD4^+ ^T cells in the tumor microenvironment may contribute to tumor development. In humanized mice which have been reconstituted with autologous DCs and implanted with tumor cells it has been shown that adoptive transfer of autologous CD4^+ ^T cells is associated with an increase in tumor mass. A high proportion of the tumor-infiltrating CD4^+ ^T cells from this model produce IL-13 and IFNγ. Moreover, blocking IL-13 prevented the rapid tumor growth associated with the addition of the CD4^+ ^T cells to the model, suggesting that in this model CD4^+ ^T cells are polarized to produce IL-13 and promote tumor development. This polarization of the CD4^+ ^T cells is mediated by DCs.

Dr. Palucka reviewed studies that explored how DCs drive the proinflammatory response in the tumor microenvironment that promotes tumor development. She noted that there are two populations of Th2 cells: regulatory Th2 cells that express IL-10, and inflammatory Th2 cells that express TNFα and IL-13, but no IL-10. The inflammatory Th2 cells are regulated by OX40L. The presence of OX40L-expressing mature DCs in the tumor microenvironment may drive the pro-inflammatory Th2 response in breast cancer. Blocking OX40L in a humanized mouse model controlled tumor development and was associated with the lack of IL-13-producing T cells within the tumor. The overwhelming majority of mature DCs that infiltrate the primary tumor express OX40L, which can drive this pro-tumor immunity.

OX40L is upregulated by thymic stromal lymphopoietin (TSLP), which is expressed by cancer cells in the tumor environment. *In vitro*, cancer cell sonicate can induce expression of OX40L on DCs. Moreover, blockade of TSLP inhibits OX40L induction and the capacity of DCs to enhance proliferation of IL-13 secreting CD4^+ ^T cells. Additionally, in the humanized mouse model, anti-TSLP controlled tumor development and was associated with reduced capacity of tumor infiltrating T cells to produce IL-13.

In summary, DC subsets elicit distinct immune responses. DCs have functional plasticity at the level of the receptor--the nature of the response of a given DC is dependent on the receptor targeted. DCs can be used for tumor immunotherapy and therapeutic vaccination, but are susceptible to signaling and regulation by the tumor microenvironment. In the development of next generation DC vaccines, we will need to improve our understanding of what is happening at the level of the tumor in order to reprogram the immune response by reprogramming DC cells. Even where new DC vaccine strategies elicit strong CTL responses, we need to enable these cells to perform within the tumor microenvironment.

## Immunotherapeutic Barriers at the Level of the Tumor Microenvironment

Thomas F. Gajewski, MD, PhD (University of Chicago) presented on the key role that the tumor microenvironment plays in determining the outcome of a tumor immune response. He noted the complexity of the tumor with respect to its structural and cellular composition and that the functional phenotypes of these cells may or may not permit an effective anti-tumor response at either the priming or effector phase. Characteristics of the tumor microenvironment may dominate during the effector phase of an anti-tumor T cell response, limiting the efficacy of current immunotherapies by inhibiting T cell trafficking into the tumor, eliciting immune suppressive mechanisms within the tumor, altering tumor cell biology and susceptibility to immune-mediated killing, or modifying the tumor stroma (i.e., vasculature, fibrosis). These features can be interrogated through pre-treatment gene expression profiling of the tumor site in individual patients; such an analysis may identify a predictive biomarker profile associated with clinical response. This strategy may also help identify biologic barriers that need to be overcome to optimize therapeutic efficacy of vaccines and other cancer immunotherapies.

Mouse models have helped to define the hallmarks of an anti-tumor response, taking into account the effector phase within the tumor microenvironment. Based on these models, a DC subset (CD8α^+^) appears necessary for priming of host CD8^+ ^T cells through cross-presentation of antigen within the draining lymph node. Antigen-specific naïve CD8^+ ^T cells that recognize the antigen within the lymph node and receive appropriate co-stimulatory and proliferative signals acquire their effector phenotype. In order to assert immune control over the tumor, these effector CD8^+ ^T cells must enter the bloodstream, and via chemokine signals, traffic to the tumor site; once there, these T cells must overcome immune regulatory/suppressive mechanisms.

In a small study of an IL-12-based melanoma vaccine, Dr. Gajewski and colleagues correlated pre-treatment biopsy gene expression to outcomes and noted that in responding patients, tumors expressed chemokines (e.g., CXCL9 which binds CXCR3 on activated CD8^+ ^T cells), which in some instances were able to recruit T cells into the tumor site. A broader transcript analysis of banked melanoma tissue demonstrated a subset of tumors with T cell markers co-associated with a panel of chemokines. Among responders in the vaccine trial, there was a pattern of expression of T cell- recruiting chemokines, T cell markers, innate immune genes, and type I IFN--all of which indicate productive inflammation.

These results were supported by other cancer vaccine studies that demonstrated a strong correlation between survival and the expression of T cell markers and chemokines within the tumors. The results from these gene expression studies may be useful in identifying biomarkers that could provide valuable information for selecting patients most likely to respond to immunotherapies. Additionally, these studies point toward specific strategies for overcoming immunologic barriers to immunotherapy at the level of the tumor microenvironment.

Thus, based on gene expression profiling, tumors can be categorized as T cell poor tumors, which lack chemokines for recruitment and have few indicators of inflammation, and T cell rich tumors, which express T cell-recruiting chemokines, contain CD8^+ ^T cells in the tumor microenvironment, and have a broad inflammatory signature. A strong presence of T cells within the tumor is predictive of clinical benefit from vaccines.

These observations prompt several important questions: 1) What dictates recruitment of activated CD8^+ ^T cells into the tumor? 2) Why are tumors with CD8+ T cells not spontaneously rejected? 3) What are the innate immune mechanisms that promote spontaneous T cell priming in a subset of patients? 4) What oncogenic pathways in tumor cells drive these two distinct phenotypes?

Studies of CD8^+ ^T cell recruitment to the tumor site point to a panel of chemokines, all of which may be produced by the melanoma tumor cells themselves. These studies suggest potential strategies to promote effector T cell migration to the tumor site that may include: direct introduction of chemokines; direct induction of chemokine production from stromal cells; eliciting local inflammation that generates chemokines (e.g., via type I IFNs, TLR agonists and possibly radiation); and altering signaling pathways in melanoma cells to enable chemokines expression by the tumor cells.

Studies that have been designed to evaluate why melanomas that attract CD8^+ ^T cells are not spontaneously rejected have pointed to several mechanisms that may exert negative regulation of T cells within the tumor microenvironment, including T cell inhibition via IDO and PD-L1, extrinsic suppression via CD4^+^CD25^+^FoxP3^+ ^Tregs, and T cell anergy due to deficiency of B7 costimulation in the tumor microenvironment.

Dr. Gajewski presented data that indicate that the immune inhibitory mechanisms present in the melanoma tumor microenvironment are driven by the CD8^+ ^T cells, not the tumor. For example, IFNγ is the major mediator for IDO and PD-L1; and CCL22 production by CD8^+ ^T cells is the major mediator for Tregs. Thus, blockade of these mechanisms may represent attractive strategies to restore anti-tumor T cell function and promote tumor rejection in patients.

To address questions underlying the mechanisms that promote spontaneous T cell priming in a subset of melanoma patients, Dr. Gajewski and colleagues used gene array data to identify markers of innate immunity that correlated with T cell infiltration. Melanoma metastases that contained T cell transcripts also contained transcripts known to be induced by type I IFNs. A knock-out mouse model demonstrated the necessity of the type I IFN axis for effective priming of a spontaneous T cell response and tumor rejection. Additional studies in knock-out mice have demonstrated that the CD8α^+ ^DC subset is responsible for this spontaneous T cell priming. In an effective anti-tumor response sensing of the tumor by a separate DC subset drives type I IFN production, which is required for CD8α^+ ^DC cross-priming of T cells. This suggests additional pathways that could be altered to promote spontaneous priming and an effective tumor response (e.g., provision of exogenous IFNβ).

In summary, there is heterogeneity in patient outcomes to cancer immunotherapies (e.g., melanoma vaccines). One component of that heterogeneity is derived from differences at the level of the tumor microenvironment. Key factors in the melanoma microenvironment include chemokine-mediated recruitment of effector CD8^+ ^T cells, local immune suppressive mechanisms, and type I IFNs/innate immunity. Understanding these aspects should improve patient selection for treatment with immunotherapies (predictive biomarker), as well as aid the development of new interventions to modify the microenvironment to better support T cell-mediated rejection of tumors.

## Regulatory Immune Cells

James H. Finke, PhD (Cleveland Clinic) presented on the biology and role of regulatory T cells and myeloid-derived suppressor cells in tumor immunology. He noted that there are two main types of Treg cells. The natural Tregs represent about 2% to 5% of cells in the peripheral blood; they differentiate in the thymus and express TCRs and CD4, as well as the α-chain for IL-2 receptor (CD25), which helps drive their proliferation. Natural Tregs also express the transcription factor FoxP3, which is critical for their function. Natural Tregs help maintain immune tolerance and inhibit autoreactive T cells; they also suppress anti-tumor immunity as shown by models that correlate Treg depletion *in vivo *with reduced tumor growth.

Inducible Tregs are the second type of CD4^+ ^regulatory T cells, which differentiate in the periphery, not the thymus. Inducible Tregs are influenced by cytokines and antigen to differentiate into either FoxP3^+ ^Tregs, Th1, Th2 or Th17 cells. Induction of this class of regulatory cells is thought to occur via TCR stimulation, IL-2 and TGFβ; MDSC and tumor cells also affect the induction of these regulatory cells.

In patients, increased numbers of tumor-infiltrating Tregs have been associated with poor prognosis for ovarian, hepatocellular, cervical, and head and neck squamous cell carcinomas. Tregs from cancer patients can suppress the *in vitro *proliferation of autologous T effector cells, a suppressive effect that can be relieved *in vitro *by diluting the Tregs.

Several mechanisms of Treg suppression have been described, including Treg production of immunosuppressive cytokines (e.g., TGFβ, IL-10), the β-galactoside binding protein galectin 1, and granzyme B. Tregs may also indirectly suppress immune functions by inhibiting DCs.

Other classes of regulatory T cells include Tr1 and Tr3 cells, which are induced by antigen. Tr1 cells secrete IL-10, whereas Tr3 cells secrete TGFβ. No specific markers for these cells have been identified, and they do not constitutively express FoxP3. Tr1 cells have been detected in some human tumors (gastric cancer and RCC). There are also CD8^+ ^Tregs, some of which express FoxP3 and secrete high levels of IL-10. Additionally, NKT regulatory cells have been described.

A number of strategies have emerged to inhibit or eliminate Tregs. They include targeting the CD25 receptor, and administration of cyclophosphamide and CpG. Cyclophosphamide has been shown to deplete Tregs and boost the efficacy of vaccines in mouse models and CpG, which targets the toll-like receptor 9, reduces FoxP3^+ ^cells in the lymph nodes of melanoma patients. Other strategies have focused on blocking Treg function, differentiation and trafficking. The receptor tyrosine kinase inhibitor sunitinib has been shown to reduce Tregs in the peripheral blood in patients with RCC and synergistically reduced Tregs in combination with a cancer vaccine in a melanoma mouse model.

Myeloid-derived suppressor cells represent another distinct class of regulatory cells. Normally present in small amounts (1% to 2% of peripheral blood cells), they accumulate under pathological conditions (~5% to as high as 25% in patients with kidney cancer). Factors produced by the tumor such as vascular endothelial growth factor (VEGF), stem cell factor (SCF), GM-CSF, G-CSF, S100A9, and M-CSF can promote the expansion of these myeloid cells and block their differentiation into DCs. Depletion of MDSCs in murine tumor models can inhibit tumor formation and metastasis and promote immune-mediated destruction of the tumor. Moreover, adoptive transfer of MDSC in murine tumor models promotes tumor growth and inhibits T cell activation.

The differentiation path of myeloid cells is dependent on the tissue environment and the growth factor milieu. In the normal environment, immature myeloid cells migrate to the peripheral organs and differentiate into DCs, macrophages and granulocytes; in the tumor microenvironment, however, the immature myeloid cells accumulate and induce T cell suppression.

MDSC expansion is mediated by a number of factors. VEGF, which is elevated in cancer and promotes tumor vascularization, induces defective differentiation of myeloid cells into DCs. SCF, IL-6 and M-CSF promote expansion of MDSCs, likely through activation of STAT3. Prostaglandin also appears to play a role in the induction of these cells. In cancer patients, GM-CSF, which is important for expansion of normal bone marrow, enhances the number of MDSCs. Indeed, GM-CSF-based vaccines may promote MDSC accumulation. Moreover, GM-CSF may promote resistance to sunitinib.

MDSCs may be activated by products from activated T cells, tumor cells or stromal cells. IFNγ, IL-4, IL-13 and products that engage toll-like receptors may all contribute to this process by activating STAT1, STAT6 or NFκB, which upregulate MDSC production of suppressive enzymes and products, including arginase, iNOS and TGFβ. Arginase reduces arginine, an amino acid required for T cell function and signaling through the TCR. Thus by depleting arginine, this enzyme arrests the cell cycle and inhibits proliferation. Both arginase and the enzyme iNOS are involved in the production of reactive oxygen species and NO, which can bind to the TCR and block its function as well as inducing apoptosis of T cells. Other suppressive mechanisms of MDSC that may play a role in tumor progression include induction of Tregs, differentiation into tumor-associated macrophages (TAMs), enhancement of a Th2 response, and downregulation of CD62L (L-selectin), a ligand involved in homing to lymph and tumor tissue.

Several approaches have been explored to target MDSCs to improve immunotherapy. These have included products that bind VEGF (i.e., VEGF-trap a fusion protein and bevacizumab), block VEGF receptor signaling (i.e., AZD2171), reduce ROS (i.e., triterpenoids) and inhibit arginase and NOS-2 expression (i.e., phosphodiesterase-5; sildanefil). Both triterpenoids and phosphodiesterase-5 have been shown to reduce MDSC function and improve T cell responses in cancer immunotherapy. Additional strategies have included all-trans retinoic acid and vitamin D3, which promote MDSC differentiation into DCs and improve T cell responses in RCC and head and neck cancer, respectively. Gemcitabine in combination with cyclophosphamide has been shown to reduce the numbers of MDSC in breast cancer. The tyrosine kinase inhibitor sunitinib, a frontline therapy for RCC, reduces MDSC levels and improves T cell function, as indicated by an increase in IFNγ production.

As previously mentioned, some MDSCs may differentiate into tumor-associated macrophages in the tumor microenvironment. There are two distinct subsets of these cells: M1 and M2 tumor-associated macrophages. M1 cells, when stimulated by LPS or IFNγ/TNF induce production of IL-1, TNF, IL-6, IL-23, IL-12, and IL-10, which can be involved in a DTH response, type 1 inflammation, Th1 responses, promoting anti-tumor activity. M2 cells, on the other hand, when stimulated with IL-4, IL-10 and IL-13 or other stimuli, produce arginase and TGFβ and other suppressive products (e.g., Il-10). M2 cells are implicated in tumor promotion, Th2 responses, allergy, and angiogenesis.

As research advances in the field, it will be useful to identify new targets for reducing Treg numbers and/or their suppressive function. It will be important to better understand the role of other immune suppressive T cell populations (Tr1/Tr3,CD8) in tumor-induced immune suppression and identify targets for blocking and/or deleting these subpopulations. Moreover, it will be beneficial to identify which of the various strategies shown to reduce MDSC in the peripheral blood of patients are also effective within the tumor microenvironment and to define which strategies promote strong anti-tumor immunity. Lastly, clinical studies are warranted to test whether effective blocking of Tregs and MDSC will provide greater efficacy for different forms of cancer immunotherapy (vaccines and adoptive therapy).

## Immune Monitoring

Lisa H. Butterfield, PhD (University of Pittsburgh) presented on immune monitoring, with the goal of defining which immune readouts correlate best with disease prognosis and clinical outcome. Peripheral blood is commonly assessed for immune parameters because it is easy to obtain at multiple time points. Whole blood can be used directly in assays or cell subsets may be separated for testing. Additionally, blood cells can be cryopreserved to allow testing at various time points. Each approach has unique advantages for particular tests. Immune assays of peripheral blood however may not reflect what is happening immunologically within a solid tumor. It should also be noted that variations in blood collection and handling (e.g., anti-coagulation additive, time and temperature since blood draw) may impact the immune assay results. The natural variations in absolute counts and percentages of particular cell types within a study population must also be considered when planning studies with immune assays to ensure that sufficient blood is drawn for all planned assays. Dr. Butterfield also discussed variation that can occur in personalized cell-based therapies, even though the same procedures and reagents were used. For example, in autologous DC vaccines, these variations can lead to patients receiving functionally different vaccines which could generate different immune responses.

Dr. Butterfield provided an example of immunomonitoring in the context of a melanoma DC vaccine clinical trial [1]. In this study, PBMC from a leukapheresis were cultured with GM-CSF and IL-4 to generate DCs, which were pulsed with the melanoma antigen, MART-1_27-35 _wild type peptide. The peptide-pulsed DC were injected into late-stage three times. The trial tested three doses (10^5^, 10^6 ^and 10^7 ^DC per injection) and two different delivery routes (intravenous and intradermal). Immune assessment was performed prior to treatment and at days 14, 28, 35, 56, and 112. Immune monitoring assays included MHC tetramer assays, ELISPOT, intracellular cytokine analysis, and cytotoxicity analysis. Among ten patients with measurable disease, one experienced a complete response after intradermal vaccination of 10^7 ^DC; two patients experienced disease stabilization with intradermal vaccination at the lower doses; seven patients experienced disease progression. Patients' immunoassay results by dose and delivery route were compared to clinical outcome. While none of the assays measuring the circulating frequency and function of the MART-1-specific T cells correlated with clinical outcome, an additional assay performed, testing the breadth of antigens responded to, was correlated to clinical outcome, and this was also observed in a follow up trial [2]. These results led to the following conclusions: 1) The MART-1- DC vaccine is safe and immunogenic; 2) MART-1-specific T cell responses are detected even at the lowest DC vaccine dose; 3) intradermal vaccination may be superior to intravenous administration; 4) in many patients the increase in circulating antigen-reactive cells is transient; and 5) complete clinical responses occurred in patients who developed T cell responses to additional class I and class II melanoma determinants (i.e., epitope spreading).

Dr. Butterfield provided an overview of the following assays that are available for assessment of immunological responses to an immunotherapy. Assays reviewed included: 1) the enzyme-linked immunosorbent assay (ELISA) for detection of antibodies; 2) indirect ELISA for detection of antigens; 3) Luminex multiplex cytokine analysis for the simultaneous measurement of multiple human cytokines, chemokines and growth factors in serum, plasma or tissue culture supernatant; 4) MHC tetramer assays, which can be combined with fluorescent Abs and flow cytometry to visualize specific T cell populations and distinguish subpopulations by phenotype and function; 5) flow cytometry, which provides information on the composition of cell populations based on expression of detectable markers, allowing monitoring and separation of distinct functional cell populations and imaging of individual cells; 6) cytotoxicity assays, including both traditional chromium release assays and novel flow cytometric assays for assessment of cell-mediated cytotoxicity; 7) ELISPOT assays, which are ELISA-based assays that detect cytokine secretion from a single cell, allowing indirect measurement of the number of cells in a sample that are producing a particular cytokine of interest, as indicated by the number of spots; 8) CFSE proliferation assays, which are used to follow dilution of a fluorescent intracellular dye and reflect on cell division (*in vitro *or *in vivo*); in combination with detection of particular markers, CFSE assays can provide information on proliferation of distinct, functional cell subpopulations (by comparison, conventional ^3^H-thymidine assays only quantify overall proliferation and do not distinguish between phenotypes); 9) delayed type hypersensitivity (DTH) reactions may be used as an *in vivo *test to gain general information on whether the patient can generate an inflammatory response to a particular Ag; because DTH responses reflect the many immune processes and variety of cells required for a response, they do not give specific information on the cells and mediators in the lesion unless biopsied; and 10) microarray gene expression assays that allow simultaneous screening of multiple genes for changes in expression in sampled tissue.

Practical considerations for designing a clinical trial with immunological monitoring were presented. The first consideration discussed was the approach for preparing blood/tissue for assays. These considerations include whether to perform assays directly on *ex vivo *samples, following overnight restimulation, after *in vitro *culture and *in vitro *stimulation, or on cryopreserved samples. Each of these approaches has implications for detection, functional assessment, and reflection of cellular activity and potential.

For studies that require assessment of tumor-specific T cells, considerations of antigen presentation are a critical component. Investigators must consider whether to use PBMC directly with whatever (limited) population of APCs are present for processing and presentation of the antigen, or generate DCs and use these as the APCs. Alternatively, the investigator may wish to reserve patients' collected cells and use a cell line as the APC for assessment of antigen-specific T cells. Each approach has advantages and limitations. For example, while direct stimulation of PBMC provides a "snapshot" of the entire mix of cells responding, antigen presentation is typically weak. While DCs are the most potent APCs and can process whole antigen, they require culturing for 5 to 7 days prior to assaying. If cell lines are used as the APCs, it is important to ensure that they are appropriately HLA matched to the patient tissue.

The investigator must also consider which population of responding cells is most appropriate to assay for the study. The study may call for assessment of the response by the whole blood, in which case total PBMCs may be appropriate. Other studies may require assessment of responses from specific cell types, requiring purification of subsets. The use of purified subsets in assays offers the advantage of making it possible to determine the source of specific activity and eliminate potentially confounding cell-cell interactions. However, the use of subsets requires testing to define purity and cells are lost during purification.

The assays selected to monitor immune responses in an immunotherapy clinical trial must be chosen based on their ability to provide reliable, meaningful answers to the scientific questions from the study. Dr. Butterfield highlighted the importance of standardized reporting of clinical immunology assays, noting that differences in reported results from an assay in the literature may reflect differences between the assays and procedures rather than just differences in the response to an immunotherapy. Guidelines designed to help improve accuracy, precision and reproducibility of many assays have been provided by CLIA (Clinical Laboratory Improvements Amendments). Dr. Butterfield described how a centralized immunology laboratory, with consistent sample handling, processing, testing and reporting, can help eliminate assay variation in clinical trials. While this approach introduces a delay in testing due to shipping of samples to the centralized lab, it supports testing of greater numbers of patients by the same procedures in larger, multi-center trials, allowing for more powerful analysis of results and recognition of trends and correlations that otherwise might not be possible.

To conclude, Dr. Butterfield summarized, that to advance innovative immunotherapy approaches, there is a need to develop and validate tools to identify patients who can benefit from a particular from of immunotherapy. Moreover, despite substantial efforts in multiple clinical trials, we do not yet know which parameters of anti-tumor immunity to measure and which assays are optimal for those measurements. The iSBTc partnered with the FDA and the NCI to create a workshop on these topics in 2009 and has prepared recommendations on immunotherapy biomarkers [3]. Additionally, iSBTc/SITC hosted a symposium on September 30, 2010 to explore issues related to biomarkers in cancer immunotherapy [4]. Presentation slides and other information about this Immuno-Oncology Biomarkers Symposium are available on the society's website [5].

## Cytokines in Cancer Immunotherapy

Kim A. Margolin, MD (University of Washington, Fred Hutchinson Cancer Research Center, Seattle Cancer Care Alliance) presented on the immunobiology of cytokines. A model of cytokine signaling was presented to demonstrate the general processes involved in cytokines binding to a receptor, causing receptor dimerization and signal transduction through receptor kinases, resulting in activation and translocation of transcription factors that influence gene expression. She noted that cytokines can be grouped into families based on similarities in receptors, with a number of cytokines sharing common receptor components (e.g., IL-2, IL-4, IL-15, 1L-9 share a common γ chain, IL-2Rγ) which may be important in signaling and in determining how the cytokine influences the innate or adaptive immune response and/or the interaction of these responses.

Dr. Margolin provided an overview of the cytokine GM-CSF, noting that it was one of first cytokines to be used in immunotherapy, not only because of its function as a hematopoietic growth factor, but also because of its ability to stimulate monocytes and macrophages. GM-CSF is produced by Th1 and Th2 cells as well as other cells, including epithelial cells, fibroblasts and tumor cells. The predominant targets for GM-CSF are cells in the macrophage-monocyte lineage, including immature DCs and myeloid progenitor cells. GM-CSF stimulates T cell immunity through effects on APCs, but may also polarize immature DCs to a suppressive or regulatory phenotype, possibly contributing to some of the negative clinical trial findings (see section on Regulatory Immune Cells above). In clinical trials, GM-CSF is not potent as a single agent; as a vaccine adjuvant and in adjuvant therapy trials for melanoma GM-CSF was not effective. However, transgenic expression of GM-CSF tumor cells has shown promise in GVAX studies, particularly in prostate cancer.

Interferons have a wide range of effects that depend on the cytokine milieu, timing and cellular interactions. Type 1 interferons (IFNα and IFNβ) signal through a shared receptor α-chain and JAK- STAT pathways. IFNα is produced by neutrophils and macrophages; IFNβ is produced by fibroblasts and epithelial cells. Type 2 interferons (IFNγ) signal through a unique receptor complex and different class of STAT molecules. Type 2 interferons are produced by T cells and NK cells. Interferons have many potent immunomodulatory effects, including upregulation of MHC class I and II expression, modulation of T and NK cell cytolytic activity (including antibody dependent cellular cytotoxicity; ADCC), modulation of macrophage and DC function, and potentially alteration of the polarization of T cell subsets within the tumor and in the circulation (e.g., decreasing Tregs and increasing Th1). Interferons also may have direct effects on tumor cells, including upregulation of MHC molecules and antiproliferative effects. These cytokines also have antiangiogenic effects, which may be associated with IFN induction of IP-10 and thrombospondin.

In clinical studies, IFN as adjuvant therapy has been shown to provide clinical benefit for patients with high-risk melanoma, improving relapse-free survival and overall survival, particularly among patients who developed signs of autoimmunity.

Interleukin-2 ("T cell growth factor") is a short chain type 1 cytokine that is structurally composed of four α-helical bundles. IL-2 is produced predominately by activated CD4^+ ^Th1 cells following TCR/CD3engagement and co-stimulation through CD28. The primary targets for IL-2 are T cells and NK cells. Many cell types express the α,β, and γ subunits of the IL-2 receptor which signals through JAK-STAT pathways as well as MAP and PI3 kinase pathways.

*In vivo*, IL-2 induces production of both type 1 and type 2 cytokines, including TNF, IFNγ, GM-CSF, M-CSF, G-CSF, IL-4, IL-5, IL-6, IL-8 and IL-10. IL-2 induces expression of its own receptor. Clinically, it causes lymphopenia, increases NK activity, and disrupts neutrophil chemotaxis. Preclinical models demonstrated IL-2 toxicities associated with capillary leak. Early clinical studies included both delivery of IL-2 and the use of IL-2 to generate LAK cells. IL-2 has also played a supportive role in a variety of adoptive T cell treatment strategies. Research conducted by the Cytokine Working Group and others have included studies on the use of IL-2, both as a single agent or in combination with other cytokines or chemotherapy, in the treatment of solid tumors and have shown benefits for patients with melanoma or renal cell cancer. On-going studies are attempting to identify approaches to limit IL-2 toxicities and identify biomarkers that can predict the subset of patients most likely to benefit from IL-2 therapy. Recent studies indicate that addition of a cancer vaccine (gp100) to high-dose IL-2 can improve survival of patients with metastatic melanoma. Future directions for IL-2 studies will include exploration of structural modifications, altering toxicity without losing activity, combinations with other agents, as well as studies to improve the understanding of mechanisms of action.

Interleukin-4 is a Th2 cytokine, whose net effect depends on the milieu. IL-4 stimulates B cells, is a growth factor for Th2 cells, and promotes proliferation and cytotoxicity of CTLs. IL-4 also stimulates MHC class II expression and enhances macrophage tumoricidal activity and contributes to DC maturation. Interleukin-4 signaling occurs through the IL-4 receptor, which is composed of the IL-4Rα and the IL-2Rγ chains, and involves JAK1, JAK3 and STAT6. Clinically, IL-4 has been studied in similar fashion to IL-2, but was found to have a less favorable therapeutic index. In the lab, however, IL-4 has taken on an important role in producing DC together with GM-CSF.

Interleukin-13 is structurally and functionally similar to IL-4. Both cytokines predominantly have inflammatory effects, favor a Th2 response and promote Ig class switching. They share a common receptor element (IL-4Rα) and either can be used together with GM-CSF to generate DCs. IL-13 differs from IL-4 however in its effect on monocytes and macrophages and its absence of effect on B and T cells.

Interleukin-6 is a pleiotropic cytokine that influences a wide array of biological activities from innate immune responses to neural development and bone metabolism. In immunity, IL-6 plays a role in B and T cell differentiation, and induction of acute phase reactants. IL-6 can be produced by a number of tumors and is associated with an unfavorable outcome in those diseases (e.g., in RCC, where it may produce thrombocytosis). IL-6 is also a growth factor for multiple myeloma. Signaling through the IL-6 receptor is mediated by JAK-STAT3 pathway.

Interleukin-7, which, like IL-15 is considered a T cell growth factor, mediates homeostatic expansion of naïve cells during lymphopenia. Signaling is through the JAK 1,3-STAT5 and the PI3-mTOR pathways. IL-7 uniquely downregulates expression of its own receptor.

Interleukin-15 is in the γ-chain cytokine family with IL-2 and IL-7 and promotes T cell growth and differentiation. IL-15 is important for recovery from leukopenia and development of a memory T cell response. IL-15 complexes with the receptor α chain on the cell of origin (DCs and monocytes), and then as a surface bound complex signals through receptors on target cells, including NK and CD8a1 cells. IL-15 promotes proliferation of NK cells, B and T cells, including memory CD8^+ ^T cells. Unlike IL-2, IL-15 does not promote proliferation of Tregs or promote activation-induced cell death, which may be advantageous for immunotherapeutic strategies.

The pleiotropic cytokine interleukin-12 mediates interactions between the innate and immune response. IL-12 receptors are present on a variety of immune cells. IL-12 induces production of IFNγ, a prototypical type I cytokine. IL-12 is also a potent inducer of counter-regulatory type 2 cytokines and has anti-angiogenic properties activities. IL-12 may find clinical application as a vaccine adjuvant as dosing and combination strategies become refined.

Interleukin-21 is a highly pleiotropic type I cytokine with effects on B cells, T cells, macrophages and DCs, and NK cells. IL-21 potentiates immunoglobulin isotype switching from IgM to IgG and enhances plasma cell differentiation and Ig production. In DCs, IL-21 increases Ag uptake and decreases Ag presentation, helping to maintain the DC in an immature form. This cytokine stimulates proliferation of CD4^+ ^T cells, NKT, and cytotoxic CD8^+ ^T cells, but not Tregs. IL-21 enhances the production of a variety of cytokines and chemokines, including IL-8 from macrophages. The IL-21 receptor includes a common IL-2R γ-chain and signaling is meditated by JAK 1,3 and STAT1, STAT3, and STAT5 pathways.

In clinical trials, IL-21 has shown promising clinical benefits among patients with melanoma and renal cancer. IL-21 may find additional clinical applications as an adjuvant or in combination with other agents.

## Immune Modulating Antibodies

James P. Allison, PhD (Memorial Sloan-Kettering Cancer Center) presented on negative regulatory mechanisms affecting T cells and the function of immune modulating antibodies in tumor immunotherapy. Dr. Allison reviewed the importance of costimulatory signals via CD28 in addition to engagement of the TCR for T cell activation. Upon concurrent TCR and CD28 signaling, the activated T cell upregulates expression of the molecule CTLA-4, which binds to the same ligand (B7-1,2) that the co-stimulatory molecule CD28 binds, but with much higher affinity. Binding of CTLA-4 to B7 has been shown to inhibit proliferation of activated T cells by disrupting cell cycling. Blockade of the CTLA-4:B7 interaction with Ab directed at CTLA-4 relieves this inhibition and allows unrestricted proliferation of activated antigen-specific T cells.

While knockout studies in mice indicate that CTLA-4 is an essential downregulator of T cell response, CTLA-4 blockade with anti-CTLA-4 antibody can be used as monotherapy to maintain an effective anti-tumor T cell response, or in combination with cancer vaccines and other treatments that kill tumor cells and thereby induce APC priming.

Initial studies in mouse models using transplantable colon carcinoma demonstrated that anti-CTLA-4 blockade led to rejection of the tumor following a brief period of tumor growth. These anti-CTLA-4-treated mice were subsequently completely immune to the tumor upon re-challenge. In models using poorly immunogenic tumors (e.g., B16 melanoma), anti-CTLA-4 alone does not control tumor growth, but in conjunction with GM-CSF tumor cell vaccine, anti-CTLA-4 synergizes to eradicate established B16 melanoma. Histological evidence indicates that anti-CTLA-4 activates the tumor vasculature and increases proliferation and infiltration of tumors by CD4^+ ^and CD8^+ ^(FoxP3^-^) effector cells, thereby increasing the ratio of effector T cells to Tregs in the tumor microenvironment. Moreover, CTLA-4 blockade also increases the ratio of IFNγ to IL-10.

A number of strategies combined with CTLA-4 blockade have been effective against poorly immunogenic tumors, including other immunotherapies and conventional therapies. Effective immunotherapies have included GVAX, peptide-pulsed DCs, DNA vaccines, depletion of CD25^+ ^cells followed by melanoma vaccination and adoptive T cell therapies. Conventional therapies that have been effective in combination with CTLA-4 blockade include chemotherapy, local irradiation, androgen deprivation, surgery, cyroablation and targeted therapies. In general, it appears that strategies that kill tumor cells or prime T cells may be enhanced by combination with CTLA-4 blockade.

To date, more that 4,000 patients have been treated with ipilimumab (anti-CTLA-4), the majority had metastatic melanoma. At the time of writing, the phase III clinical trial data that showed improved survival in second line treatment of metastatic melanoma with ipilimumab was under review at the FDA; a phase III trial with ipilimumab as first line melanoma treatment has been completed and is awaiting data analysis. CTLA-4 blockade has also been tested in phase II clinical trials of castrate-resistant prostate cancer and has led to objective responses in patients with ovarian, lung and kidney cancer.

Using RECIST or modified WHO response criteria, the response rate with ipilimumab monotherapy for melanoma has been approximately 15%, with some complete responses. CTLA-4 blockade has provided approximately 35% to 40% survival benefit. For many patients, the response is durable, allowing the benefit to be maintained months to years without retreatment. Dr. Allison briefly summarized these results and mentioned how, with the continued growth of the tumor often observed following ipilimumab treatment, many patients are originally classified as non-responders, some may even have evidence of progressive disease, even though anti-CTLA-4 treatment may lead to subsequent elimination of the tumor.

Ipilimumab treatment in humans has been associated with adverse events, including colitis, rashes, hypophysitis, and hepatitis, most of which resolve with symptomatic treatment and cessation of therapy, but may require steroid administration.

Phase III clinical trial results of ipilimumab treatment of metastatic melanoma demonstrated that ipilimumab (with or without a gp100 peptide vaccine) provided a significant increase in overall survival rate compared to gp100 alone. At one year, the survival rate was 44% (ipilimumab + gp100) and 46% (ipilimumab alone), compared to 25% for treatment with gp100 alone. The survival advantage with ipilimumab persisted, with survival at year two at 22% (ipilimumab + gp100) and 24%% (ipilimumab alone), compared to 14% for treatment with gp100 alone. Moreover, melanoma survival outcomes with 10mg/kg ipilimumab maintenance dosing may be higher than with 3 mg/kg single dosing, with 2 year survival over 55% in one study.

Dr. Allison presented a case in which ipilimumab in combination with GM-CSF-transfected autologous tumor cells (GVAX) was used to treat ovarian cancer. This combination treatment led to a delayed reduction in CA-125, a serum marker of ovarian cancer, as well as reduction in the tumor load. Repeated combination treatment upon rebound of CA-125 over seven years has helped control the tumor and demonstrates the lack of tumor resistance to CTLA-4 blockade.

These observations point to critical questions for further clinical development of anti-CTLA-4 regarding the cellular and molecular mechanisms involved in the anti-tumor effects, the characteristics that distinguish responders from non-responders, and the best combination strategies to improve cancer outcomes.

Dr. Allison reviewed a class of tumor markers known as the cancer testis (CT) antigens. These are expressed during germ cell development and in many cancer types but not in other normal tissue. Spontaneous immune responses against CT antigens can be detected in some cancer patients. Indeed, (pre-existing or induced) antibody to the antigen NY-ESO-1 may correlate with clinical response to ipilimumab treatment and may play a role in predicting which patients are most likely to benefit from treatment. These antibody responses were also reflected in T cell responses to these antigens. Another marker that may be useful in predicting clinical benefit from ipilimumab is the CD4^+ ^T cell marker ICOS, a member of the B7-CD28 family.

Another member of the B7-CD28 family, PD-1 involves a distinct inhibitory pathway to inhibition of T cells in the tumor microenvironment and represents a potential target for therapy alone or in combination with CTLA-4 blockade. Blockade of one receptor leads to reciprocal upregulation of the other. Moreover, each receptor plays a distinct but pro-suppressive role in regulatory T cells. In a phase II clinical trial of anti-PD-1 in 21 patients with diverse cancers, one patient experienced complete response and five experienced partial responses with limited toxicity. In a mouse model, blockade of the PD-1 pathway was shown to synergize with CTLA-4 blockade in reducing tumor volume and improving overall survival.

B7-H3 and B7x (B7-H4) represent another set of potential targets from the B7-CD28 family for combination treatment. These molecules are not found in the hematopoietic system, but are upregulated in certain cancers, including human prostate cancer. B7-H3 and B7x inhibit effector functions via pathways distinct from CTLA-4 and PD-1 by downregulating cytokine production.

Additional molecular targets that may warrant further exploration in combination treatment include Ox40 (CD134), which is present primarily on CD4^+ ^T cells, and 4-1BB (CD137), which is mainly on CD8^+ ^T cells. These are both members of the TNF receptor family and are expressed on activated T cells. Antibodies to these molecules are agonists, increase T cell survival and proliferation, and have anti-tumor effects alone or in combination with vaccines in animal models.

## Cancer Vaccines

Co-organizer of the 2010 Primer on Tumor Immunology and Biological Therapy of Cancer™ Walter J. Urba, MD, PhD (Earle A. Chiles Research Institute) presented on cancer vaccines, providing an overview of the long history and current strategies under investigation. Dr. Urba discussed attempts as early as 1777 and 1808 to induce responses through inoculation of cancer cells. By the late 1960s Nadler and Moore had demonstrated the potential benefit of immunotherapy of a variety malignant diseases by transfusing lymphocytes from therapeutic donors who had been inoculated with the patient's tumor cells [6]. With our much more advanced understanding of molecular and cellular processes involved in generating a productive anti-tumor response, we are now able to design cancer vaccine strategies that take into consideration the importance of specific immune cell subsets and tumor antigens, co-stimulation, negative regulation and tumor cell biology. As we improve our understanding of tumor immunology and combine and test various strategies, we will continue to improve the effectiveness of cancer vaccines. The success of these strategies, however, will depend on our ability to identify appropriate tumor-rejection antigens and to stimulate a potent immune response, which in turn requires selecting the proper adjuvant, generating the correct type of immune response and eliciting long-term immunological memory. Moreover, effective cancer vaccines should be designed to minimize the risk of autoimmunity and prevent immune evasion by the tumor cells. Effective vaccines must overcome escape mechanisms exploited by tumor cells (e.g., antigen loss, expression of immunosuppressive cytokines, induction of Tregs and MDSC) as well as the tumor's capacity to affect T cell trafficking and normal immune regulation (e.g., via CTLA-4 and PD-1). In addition, an effective strategy must provide clinical benefit in the context of an aging immune system reflective of the patient population for the particular malignancy.

Simply stated, a cancer vaccine teaches the immune system to recognize tumor cells. The vaccine consists of three components: 1) a tumor antigen, 2) an adjuvant, and 3) an immune modulator. Hundreds of tumor antigens are known, including tumor-specific antigens, antigens from new fusion proteins, antigens resulting from mutations, differentiation antigens, overexpressed antigens, and viral antigens (Table 1).

**Table 1:** Human Tumor Antigens

**Shared tumor-specific antigens **(Cancer - testis antigens)	MAGE, BAGE, GAGE, GnTV, NY-ESO-1, RAGE, TRP2-INT2
**Antigens from new fusion proteins**	bcr-abl, ETV6/AML

**Antigens resulting from mutations**	BRAF, CDK4, β-catenin, CASP-8, K-ras, hsp70-2, EF2, TPI, Cdc27, p53

**Differentiation antigens**	Tyrosinase, Melan-A ^MART1^, gp100, gp75^TRP1^, TRP2, CEA, PSA, PAP, PMSA

**Overexpressed antigens**	P53, HER2-neu, PRAME, survivin, telomerase, WT-1

**Viral antigens**	HPV16 E7, EBV

**Other antigens**	MUC-1, Idiotype

Mutation discovery experiments have indicated that a single tumor may contain as many as 1,300 unique somatic mutations, representing hundreds of potential antigens. The National Cancer Institute has recently published a prioritized list of cancer vaccine target antigens based on predefined and preweighted objective criteria [7]. From this pilot project characteristics of an ideal tumor antigen for vaccine development included consideration of the following criteria of the antigen (ranked by perceived importance): 1) therapeutic function, 2) immunogenicity, 3) role of the antigen in oncogenicity, 4) specificity, 5) expression level and percent antigen-positive cells, 6) stem cell expression, 7) number of patients with antigen-positive cancers, 8) number of antigenic epitopes and 9) cellular location of expression. Based on these predefined and preweighted criteria, the following tumor antigens were prioritized in rank order: WT1, MUC1, LMP2, HPV E6 E7, EGFRvIII, HER-2/neu, Idiotype, MAGE A3, p53 nonmutant, and NY-ESO-1.

Antigens for active immunization can be administered to patients in a number of forms, including as antigenic peptides (short and long), whole proteins or virus-like particles (with adjuvants, combined with lipids or liposomes, with gp96, Hsp70, or Hsp90). Recombinant viruses (e.g., adenovirus, fowlpox virus, vaccinia virus) containing tumor antigen genes may be used. Naked DNA encoding tumor antigen genes may be injected. Recombinant bacteria containing tumor antigen genes may also be used to deliver the antigen. Alternatively, patients may be transfused with cells expressing tumor antigens (dendritic cells pulsed with antigen, modified or unmodified tumor cells).

Adjuvants are commonly included in vaccines to increase immunogenicity. There are no standard approaches for defining optimal adjuvanticity other than measures of immune response or clinical response in a large trial. Adjuvants may play a role in activation and recruitment of professional APCs or induction of a cytokine milieu to support a Th1 (or Th2, or Th17) response. Adjuvants may also promote innate immunity or provide an antigen depot effect. Immunotherapies have included a variety of adjuvants alone or in combination, including alum, BCG (Bacillus Calmette-Guérin; attenuated *Mycobacterium bovis*), incomplete Freund's adjuvant, cytokines (e.g., IL-12, GM-CSF, IFN-alpha), Toll-like receptor agonists (e.g., TLR3, 4, 7, 8, 9), and saponins.

Cancer vaccine strategies have included both prophylactic and therapeutic vaccines. Prophylactic vaccines have been developed or are being tested for a variety of infectious agents implicated in oncogenesis, including bacteria (e.g., *Helicobacter pylori *for stomach cancer), viruses (e.g., HPV for cervical cancer; hepatitis B and C for liver cancer) and parasites (e.g., schistosomes for bladder cancer).

Early therapeutic cancer vaccine trials provided disappointingly poor responses. More recent studies however have demonstrated a clinical benefit of cancer vaccines using defined antigens. Examples include regression of cervical neoplasia with HPV vaccine, promising results for an idiotype lymphoma vaccine, increased response rate and PFS in melanoma with gp100 peptide vaccine in combination with high dose IL-2, improved survival with PROSTVAC-VF, and improved survival of patients with metastatic hormone-refractory prostate cancer following treatment with the recently FDA-approved DC vaccine Sipuleucel-T.

Co-stimulatory molecules have been included in cancer vaccines. Candidate immune modulators that have been used include B7-1, ICAM-1, LFA-3, as well as a fusion protein of these three molecules, TRICOM, which dramatically increases T cell activation, and in conjunction with GM-CSF and a PSA vaccine (PROSTVAC) significantly increased overall survival of patients with metastatic prostate cancer compared to controls [8].

Dr. Urba concluded his presentation with a summary of the clinical trial results that eventually led to the FDA approval of the first therapeutic cancer vaccine, Sipuleucel-T, for treatment of prostate cancer. Future active vaccination strategies will be able to take advantage of our growing understanding of a variety of tumor antigen targets, delivery systems, signaling and interactions in the tumor microenvironment, as well as optimized adjuvants and immune modulators that will increase efficacy of cancer vaccines.

## Adoptive T Cell Therapy

Patrick Hwu, MD (MD Anderson Cancer Center), co-organizer of the 2010 Primer, provided an overview of adoptive T cell therapy. In this therapy, T cells are removed from the patient, manipulated, and expanded *in vitro *before being transferred back to the patient. *In vitro *expansion avoids the suppressive immunoregulatory environment present in cancer patients and allows use of T cells from donors. *Ex vivo *expansion increases the number of antigen-specific T cells and provides the opportunity to carefully control the subset and phenotype of cells that are infused with respect to antigen specificity and activation state.

Evidence in support of the effectiveness of T cell therapy has come from several different approaches. Dr. Hwu summarized results in which virus-specific CTLs were expanded to prevent viral infection post-transplant (EBV and CMV). Another line of evidence that has demonstrated the effectiveness of adoptive T cell therapy has come from donor lymphocyte infusion (DLI), which has been shown to enhance graft versus tumor effect in chronic and acute myeloid leukemia, acute lymphocytic leukemia, and myelodysplasia. Additional evidence for adoptive T cell strategies has come from treatment of melanoma with tumor-infiltrating lymphocyte (TIL) therapy.

A variety of regulatory immune cells have been shown to suppress tumor immunity as discussed earlier. Depletion of these regulatory cells with chemotherapy prior to T cell transfer allows the infused T cells to persist longer *in vivo*, where they can exert their anti-tumor effect and promote a clinical response. While adoptive T cell therapy has shown promise for treatment of cancer, this therapeutic approach has inherent challenges. It is a rigorous therapy that requires excellent performance status. The tumor must be accessible to generate TILs. Unfortunately, adequate numbers of tumor-specific T cells are only generated in approximately 40% - 50% of patients. Moreover, *in vitro *generation of T cells the may require 4 - 6 weeks, and once infused, migration of the T cells to the tumor may be suboptimal.

To address these challenges and improve adoptive immunotherapy, investigators have developed new strategies to enhance *in vitro *generation and *in vivo *persistence of T cells, to improve migration of infused cells to the tumor, and to improve recognition of the tumor by the transferred T cells. Recent developments with "young TIL" therapy have enhanced T cell expansion, allowing more rapid and successful T cell generation. New approaches to improve *in vivo *persistence of infused T cells have included co-transfusion of T cells together with DCs, and lymphodepletion prior to infusion. This has not only extended T cell survival, but has also been associated with improvements in objective response in therapy of patients with metastatic melanoma. Migration of infused T cell to the tumor has been improved by transducing the chemokine receptor CXCR2 to the T cells prior to expanding the cells *in vitro*. This receptor binds chemokines secreted by the tumor that serve as autocrine growth factors. In addition to transducing chemokines receptors to aid T cell trafficking to the tumor, investigators have inserted native TCR genes to improve recognition of the tumor. This offers the advantage of being able to use T cells from peripheral blood, not just TILs, and in cases where the tumor does not harbor sufficient T cell infiltrates. Chimeric antigen receptors (CARs) have been used to enhance T cell activation and costimulation. These hybrid receptors recognize the corresponding tumor antigen in a non-MHC-restricted manner and, upon binding, elicit cytokine production and cytotoxicity of the T cell. The costimulatory moiety promotes enhanced T cell persistence and a sustained anti-tumor reaction.

In summary, the infusion of antigen-specific T cells can be effective in patients to induce tumor regression and decrease viral infections post-transplant. T cell therapy can be more potent than cytokine or vaccine therapy, possibly because expansion and activation of T cells can be controlled in the laboratory in a non-immunosuppressive environment. Future studies will rely on rational combinations of adoptive therapy with active immunization, as well as with other immune adjuvants and targeted therapies. Prospective, randomized studies of adoptive T cell therapy are warranted to optimize this therapy and improve outcomes of patients with cancer.

In conclusion, the 2010 Primer on Tumor Immunology and Biological Therapy of Cancer™ provided a comprehensive overview of the principles of immunology, with the aim of advancing the understanding and application of immunotherapy to improve cancer outcomes. Presentation slides and a Primer webinar can be viewed at the iSBTc/SITC website [9].

## Competing interests

The authors declare that they have no competing interests.

## Authors' contributions

PH and WU co-planned, co-organized, and co-chaired the Primer. JB drafted the manuscript. PH, WU and FM critically reviewed and WU edited the manuscript. All authors read and approved the final manuscript.

## References

[B1] Butterfield LH Ribas A Dissette VB Amarnani S Vu H Oseguera D Wang HJ Elashoff RM McBride WH Mukherji B Cochran A Glaspy JA Economou JS Determinant spreading associated with clinical response in dendritic cell-based immunotherapy for malignant melanoma Clin Cancer Res 2003 9 998 1008 12631598

[B2] Ribas A Glaspy JA Lee Y Dissette VB Seja E Vu H Tchekmedyian NS Oseguera D Comin-Anduix B Wargo JA Amarnani SN McBride W Economou JS Butterfield LH Role of dendritic cell phenotype, determinant spreading and negative costimulatory blockade in dendritic cell-based melanoma immunotherapy J Immunother 2004 27 354 367 10.1097/00002371-200409000-00004 15314544

[B3] Butterfield LH Palucka AK Britten CM Dhodapkar MV Hakansson L Janetzki S Kawakami Y Kleen TO Lee PP Maccalli C Maecker HT Maino VC Maio M Malyguine A Masucci G Pawelec G Potter DM Rivoltini L Salazar LG Schendel DJ Slingluff CL Jr Song W Stroncek DF Tahara H Thurin M Trinchieri G van Der Burg SH Whiteside TL Wigginton JM Marincola F Khleif S Fox BA Disis ML Recommendations from the iSBTc-SITC/FDA/NCI Workshop on Immunotherapy Biomarkers Clin Cancer Res 2010 in press 10.1158/1078-0432.CCR-10-2234 PMC3096674 21558394

[B4] Butterfield LH Disis ML Khleif SN Balwit JM Marincola FM Immuno-Oncology Biomarkers 2010 and Beyond: *Perspectives from the iSBTc/SITC Biomarker Task Force* J Transl Med 2010 8 1 130 10.1186/1479-5876-8-130 21138581 PMC3014892

[B5] Biomarkers Symposium Slides http://www.isbtc.org/meetings/am10/biomarkers10/65

[B6] Nadler SH Moore GE Immunotherapy of Malignant Disease AMA Arch Surg 1969 99 3 376 381 5798432 10.1001/archsurg.1969.01340150084016

[B7] Cheever MA Allison JP Ferris AS Finn OJ Hastings BM Hecht TT Mellman I Prindiville SA Viner JL Weiner LM Matrisian LM The Prioritization of Cancer Antigens: A National Cancer Institute Pilot Project for the Acceleration of Translational Research Clin Cancer Res 2009 15 5323 5337 10.1158/1078-0432.CCR-09-0737 19723653 PMC5779623

[B8] Kantoff PW Schuetz TJ Blumenstein BA Glode LM Bilhartz DL Wyand M Manson K Panicali DL Laus R Schlom J Dahut WL Arlen PM Gulley JL Godfrey WR Overall Survival Analysis of a Phase II Randomized Controlled Trial of a Poxviral-Based PSA-Targeted Immunotherapy in Metastatic Castration-Resistant Prostate Cancer J Clin Oncol 2010 28 1099 1105 10.1200/JCO.2009.25.0597 20100959 PMC2834462

[B9] Primer Slides http://www.isbtc.org/meetings/am10/primer10/44

